# Comparative Susceptibility of *Aedes albopictus* and *Aedes aegypti* to Dengue Virus Infection After Feeding on Blood of Viremic Humans: Implications for Public Health

**DOI:** 10.1093/infdis/jiv173

**Published:** 2015-03-17

**Authors:** James Whitehorn, Duong Thi Hue Kien, Nguyet Minh Nguyen, Hoa L. Nguyen, Peter P. Kyrylos, Lauren B. Carrington, Chau Nguyen Bich Tran, Nguyen Thanh Ha Quyen, Long Vo Thi, Dui Le Thi, Nguyen Thanh Truong, Tai Thi Hue Luong, Chau Van Vinh Nguyen, Bridget Wills, Marcel Wolbers, Cameron P. Simmons

**Affiliations:** 1 London School of Hygiene and Tropical Medicine; 2 Oxford University, United Kingdom; 3 Oxford University Clinical Research Unit; 4 Hospital for Tropical Diseases, Ho Chi Minh City, Vietnam; 5 University of Melbourne, Australia

**Keywords:** Dengue, *Aedes aegypti*, *Aedes albopictus*, susceptibility, transmission

## Abstract

*Aedes albopictus* is secondary to *Aedes aegypti* as a vector of dengue viruses (DENVs) in settings of endemicity, but it plays an important role in areas of dengue emergence. This study compared the susceptibility of these 2 species to DENV infection by performing 232 direct blood-feeding experiments on 118 viremic patients with dengue in Vietnam. Field-derived *A. albopictus* acquired DENV infections as readily as *A. aegypti* after blood feeding*.* Once infected, *A. albopictus* permitted higher concentrations of DENV RNA to accumulate in abdominal tissues, compared with *A. aegypti*. However, the odds of *A. albopictus* having infectious saliva were lower than the odds observed for *A. aegypti* (odds ratio, 0.70; 95% confidence interval, .52–.93). These results quantitate the susceptibility of *A. albopictus* to DENV infection and will assist parameterization of models for predicting disease risk in settings where *A. albopictus* is present.

**(See the editorial commentary by Christofferson on pages 1177–9.)**

Dengue is the most important arboviral infection of humans, with an estimated 00 million clinically apparent infections annually [1, 2]. In dengue-endemic countries, *Aedes aegypti* (Linnaeus) is widely accepted to be the primary vectors of dengue viruses (DENVs), with *Aedes albopictus* (Skuse) regarded as a secondary vector. Yet *A. albopictus* can clearly transmit DENV at scales that are important to public health. For example, outbreaks of dengue during 1976–1977 (in the Seychelles), 2004 (in Ningbo, China), 2007 (in Gabon), 2009 (in Mauritius), 2010 (in Dongguan, China), and 2014 (in Guangdong, China, and Japan) were associated with *A. albopictus* [3–10]. These outbreaks demonstrate that, although *A. albopictus* can be a competent vector of DENV, there must be biological features that render *A. albopictus* generally less well equipped to transmit DENV, compared with *A. aegypti*.

*Aedes albopictus* originated in Asia but has geographically spread through global trade, particularly via used car tires infested with *A. albopictus* eggs and larvae [11]. It is now distributed throughout the United States, Central America, and South America and in temperate African and European countries [12–14]. Numerous features have supported the expansion of *A. albopictus,* including the ability of the eggs to undergo diapause [15]. There is also speculation around their ability to outcompete established species of mosquitoes [16, 17].

The expansion of *A. albopictus* has led to concern of an associated increase in the range of dengue transmission. Lambrechts et al reviewed vector competence literature and reported that, although *A. albopictus* were more susceptible to DENV midgut infection, rates of virus dissemination to other tissues were significantly lower in *A. albopictus* than in *A. aegypti* [18]. Additionally, laboratory-reared *A. albopictus* became increasingly susceptibility to DENV, which may have been a confounding variable in the literature. Furthermore, the comparative vector competence literature has been derived entirely from laboratory experiments using artificial blood meals and laboratory-passaged viruses. How well these laboratory conditions replicate the pathogenesis of DENV transmission from viremic humans to mosquitoes in the field is uncertain.

The benefits of understanding why *A. albopictus* is largely a secondary vector of DENV in settings of endemicity are numerous. First, existing risk-analysis models of the likelihood of dengue outbreaks in southern Europe and the United States could be improved with quantitative estimates of *A. albopictus* vector competence [19, 20]. Second, probability maps of dengue occurrence could be refined with better estimates of the relative vector competence of *A. albopictus* versus *A. aegypti.* Third, if the historical literature is correct and *A. albopictus* is more resistant than *A. aegypti* to disseminated DENV infection*,* then this provides an opportunity to identify species-specific antiviral defense mechanisms. Last, novel dengue control efforts using *Wolbachia*-infected *A. aegypti* will be better informed with an understanding of the vector competence of *A. albopictus* in candidate intervention settings [21]. To these ends, the current study compared the susceptibility of *A. aegypti* and *A. albopictus* to initial and disseminated DENV infection after direct blood-feeding episodes on viremic patients with dengue.

## METHODS

### Ethics Statement

All participants provided written informed consent to participate in the study. The study protocols were reviewed and approved by the Scientific and Ethical Committee of the Hospital for Tropical Diseases (CS/ND/12/15) and the Oxford Tropical Research Ethical Committee (OxTREC 29–12). All investigations were conducted in accordance with the principles expressed in the Declaration of Helsinki.

### Patient Cohorts

The study was performed at the Hospital for Tropical Diseases in Ho Chi Minh City, Vietnam, between September 2012 and November 2013. The inclusion criteria were age of ≥15 years, fever duration of ≤96 hours, and clinical suspicion of dengue; positive result of an NS1 rapid test; and provision of written informed consent. The exclusion criteria were current pregnancy, determined by clinical examination or a urine dipstick test for β-human chorionic gonadotropin; current intensive care unit stay; intellectual disability; a history of severe reactions to mosquito bites; and severe dermatological conditions. Demographic and clinical information were recorded prospectively in a standard case report form.

### Mosquitoes for Blood-Feeding Experiments

All of the mosquitoes (*A. aegypti* and *A. albopictus*) that fed on patients with dengue were F3 generation and derived from 2 independent pooled larval collections, each sampled from 3 locations within 5 km of each other in Ho Chi Minh City. The *A. aegypti* used were distinct from those in our previous study [22]. Briefly, field-caught larvae were pooled and fed commercial dry fish and dog food. Mosquitoes were housed as described previously [22]. Briefly, adults (F1 generation) were kept in cages containing males and females in an environmental chamber with 12-hour cycles of light and dark at 27°C and 70% relative humidity. F1 females were provided blood meals by direct feeding on afebrile healthy human volunteers for multiple gonotrophic cycles over 45 days, with 15% sucrose provided freely in addition. Eggs from F1 females were hatched and reared and the subsequent F2 females provided with human blood meals as described above. When female F2 mosquitoes were 12 days old, they were killed and pooled into groups of 10 mosquitoes. Each pool was homogenized and tested, along with appropriate controls, by reverse transcription–polymerase chain reaction (RT-PCR) to confirm the absence of DENV, Japanese encephalitis virus, and chikungunya virus. Eggs collected and stored from F2 females were the source of F3 females that were used for direct feeding on patients with dengue.

### Experimental Exposure of Patients to Mosquitoes

Each patient was assigned a schedule of 2 exposures to mosquitoes on 2 different study days during the first 4 days after enrollment. The patient's forearm was exposed to 30–40 *A. aegypti* and *A. albopictus* aged 3–7 days (using a 2:3 ratio of *A. aegypti* to *A. albopictus* because preliminary experiments indicated that a higher fraction of *A. aegypti* took a blood meal) contained in a mesh-covered plastic cup that was held against the patient's forearm for 5 minutes. After 5 minutes, mosquitoes were returned to the insectary and subjected to cold anesthesia at 4°C for 45 seconds. Engorged mosquitoes were transferred to 500-mL plastic cups, separated by species, and maintained in an environmental chamber with 12-hour cycles of light and dark at 27°C and 70% relative humidity for 14 days. The number of dead mosquitoes was recorded daily.

### Clinical Adverse Events

Postexposure severe adverse events were defined as any event that was clinically significant (ie, requiring clinical intervention, prolonged hospitalization, or admission to an intensive care unit) and possibly, probably, or definitely related to experimental exposure to mosquitoes.

### Detection of DENV in Mosquito Tissues and Saliva

Technicians blinded to clinical and virological details of the participants performed laboratory assays of the mosquitoes. Mosquitoes were killed by cold exposure. The abdomen was dissected from the rest of the mosquito body and suspended in 0.5 mL of mosquito diluent (2% [v/v] heat-inactivated fetal calf serum [FCS] in Roswell Park Memorial Institute 1640 medium, antibiotics, and antimycotics). Individual mosquito abdomens were homogenized with 1-mm zirconia/silica beads for 15 minutes at 30 Hz by using a TissueLyser II system (Qiagen), as described previously [22]. Mosquitoes were scored as being infected with DENV, using a previously described quantitative RT-PCR analysis of homogenized tissue, with the results expressed as copies per abdomen [23]. Infectious virus in the saliva of individual mosquitoes was detected as described previously [22]. Briefly, the proboscis of dewinged and delegged mosquitoes was inserted into the end of a micropipette tip containing 6 µL of filtered saliva medium (a 1:1 solution of 15% [v/v] sucrose in normal saline and inactivated FCS). After 30 minutes, the 6 µL of saliva medium was ejected and then drawn into a pointed glass capillary tube (tip diameter, <0.3 µm), after which the volume of saliva medium derived from 1 mosquito was injected into the thorax of 4–6 *A. aegypti* (age, 4–7 days; volume injected, approximately 1 µL per mosquito). These injected mosquitoes were maintained for 7 days in an environmental chamber as described above. After 7 days, the mosquitoes were killed and the bodies pooled, homogenized, and tested by quantitative RT-PCR for DENV infection, with saliva samples scored as positive or negative depending on this result.

### Sequence Amplification and Sequencing of the Gene Encoding DENV Envelope Protein

Viral RNA was extracted as previously described from plasma and mosquito abdomen tissues [22]. Complementary DNA (cDNA) was synthesized from 8 µL of viral RNA by using the Superscript III First-Strand Synthesis System for RT-PCR according to manufacturers’ instructions. The genomic region spanning the genes encoding premembrane and envelope proteins was amplified from the cDNA in 16 amplimers. Subsequent steps were performed according to the manufacturers instructions for 454 GS-Junior Next Generation Sequencing method (Roche). Briefly, the primers in this first PCR round contained universal tails at the 5′ end to allow the addition of 454 sequencing-specific nucleotides and isolate-specific multiplex identifiers (also known as “barcodes”) in a second PCR round. The first-round and second-round PCR analyses used FastStart High Fidelity polymerase (Roche). The long-read sequencing performance of the 454 GS-Junior (between 400 and 500 bases), in combination with a sample pooling strategy that uses barcoded amplicons, was used for parallel analysis of pooled samples. GS Mapping software was used for primer trimming and alignment of reads against a reference sequence DENV-1/VN/BID-V2732/2007 (GenBank accession number GQ199773.1). Sequence quality was measured using Phred (Q) scores with a minimum acceptable threshold of 95% of sequencing reads having Q scores of >20 (1/100 errors per base).

### Dengue Diagnostic Tests

Serological responses were detected using immunoglobulin M (IgM) and immunoglobulin G (IgG) antibody-capture enzyme-linked immunosorbent assays in accordance with the manufacturer's instructions (Panbio, Australia). In accordance with the manufacturer's instructions, we classified serological profiles as “probable secondary” when >22 U of IgG were detected in either acute or early convalescent samples. If acute or early convalescent samples were IgM positive but IgG negative, serological profiles were classified as “probable primary.” When IgM or IgG tests result were equivocal, we classified the serological profile as “indeterminate.”

DENV plasma viremia levels were measured by a validated quantitative RT-PCR assay that has been described previously [23].

### Statistical Analysis

The probability of successful human-to-mosquito transmission was compared between *A. aegypti* and *A. albopictus*, using marginal logistic regression models. Models for assessment of abdomen samples were adjusted for the patient's log_10_-transformed viremia level, and models for assessment of saliva samples were further adjusted for the abdominal tissue viremia level. Analyses were performed for all patients and stratified by serotype. To account for potential within-patient correlation, model-robust sandwich standard error estimates were used throughout to construct confidence intervals (CIs) and *P* values. We derived 50% mosquito infectious doses (MID_50_ values; defined as plasma viremia levels corresponding to a 50% probability of infection) for abdomen infection on the basis of marginal logistic regression coefficients for each mosquito type, and corresponding 95% CIs were calculated using the delta rule. Since the proportion of mosquitoes with infectious saliva was <50%, MID_50_ values were not estimated. In the initial analysis of the transmission of DENV to saliva, mosquitoes with uninfected abdomens were assigned a log value of 0. This analysis was also run with exclusion of mosquitoes with uninfected abdomen's. The relationship between various covariates and the probability of successful human-to-mosquito transmission was assessed for each mosquito type, using a similar multivariable marginal regression model. The covariates assessed were day of illness, plasma viremia level, serotype, serological result, and abdominal viral burden. The number of mutations in the DENV-1 consensus sequence was compared between mosquito types, using a stratified version of the Wilcoxon test with stratification by patient [24]. All the analyses were performed using the R statistical software package, version 2.13.2 (R Foundation for Statistical Computing; Vienna, Austria).

## RESULTS

### Study Population Characteristics

Between September 2012 and November 2013, 120 patients with dengue were enrolled and experimentally exposed to field-derived *A. aegypti* and *A. albopictus* on 2 randomly allocated days within the first 4 study days. The patient enrollment flowchart is shown in Supplementary Figure 1. The final cohort for analysis comprised 118 DENV viremic patients with 232 independent mosquito exposure events. The baseline characteristics of the patients enrolled are shown in Table 1. Experimental exposure to mosquitoes was well tolerated, and no patient experienced a severe adverse event or required withdrawal from the study. DENV serotype 1 (DENV-1) and DENV-4 were responsible for 26% and 44% of cases, respectively, with DENV-2 (13%) and DENV-3 (16%) also represented.

**Table 1.** Characteristics of 118 Patients With Dengue Virus (DENV) Infection, by DENV Serotype

Characteristic	DENV-1 (n = 31)	DENV-2 (n = 15)	DENV-3 (n = 19)	DENV-4 (n = 52)	Overall (n = 118)^a^
Age, y	23 (19–31)	23 (21–32)	25 (20–27)	27.5 (22–34)	26 (20–32)
Sex					
Male	12 (38.7)	7 (46.7)	5 (26.3)	17 (32.7)	41 (34.8)
Female	19 (61.3)	8 (53.3)	14 (73.7)	35 (67.3)	77 (65.3)
Illness duration at enrollment, d^b^					
1	0 (0)	0 (0)	0 (0)	0 (0)	0 (0)
2	7 (22.3)	5 (33.3)	0 (0)	7 (13.4)	19 (16.2)
3	11 (36.7)	3 (20.0)	7 (36.8)	20 (38.4)	42 (35.9)
4	12 (40.0)	7 (46.7)	11 (57.9)	24 (46.2)	54 (46.2)
5	0 (0)	0 (0)	1 (5.3)	1 (2.0)	2 (1.7)
Viremia level, log_10_ copies/mL	8.1 (6.9–8.7)	7.6 (7.1–8.9)	6.6 (5.5–7.1)	7.2 (6.5–7.8)	7.2 (6.6–8.1)
Serological profile^c^					
Primary	10 (32.3)	0 (0)	1 (5.3)	1 (2.0)	12 (10.3)
Secondary	17 (54.8)	12 (80.0)	16 (84.2)	48 (94.1)	94 (80.3)
Indeterminate	4 (12.9)	3 (20.0)	2 (10.5)	2 (3.9)	11 (9.4)
Clinical classification					
Dengue	26 (83.9)	14 (93.3)	16 (84.2)	47 (90.4)	104 (88.1)
Dengue with warning signs	5 (16.1)	1 (6.7)	2 (10.5)	4 (7.7)	12 (10.2)
Severe dengue	0 (0)	0 (0)	0 (0)	0 (0)	0 (0)
Others	0 (0)	0 (0)	1 (5.3)^d^	1 (1.9)^e^	2 (1.7)
Transferred to ICU	1 (3.2)	0 (0)	0 (0)	0 (0)	1 (0.9)

Data are no. (%) of patients or median value (interquartile range).

Abbreviation: ICU, intensive care unit.

^a^ The viremia level for 1 patient was below the limit of detection.

^b^ Data for 1 patient were missing.

^c^ Data for 1 patient with DENV-4 infection were missing.

^d^ One patient was categorized as having a “viral infection” but was actually infected with DENV-3.

^e^ One patient was categorized as having a “viral infection” but was actually infected with DENV-4.

### Susceptibility of *A. aegypti* and *A. albopictus* to Acquisition of DENV Infection After Direct Feeding

**Figure 1.**
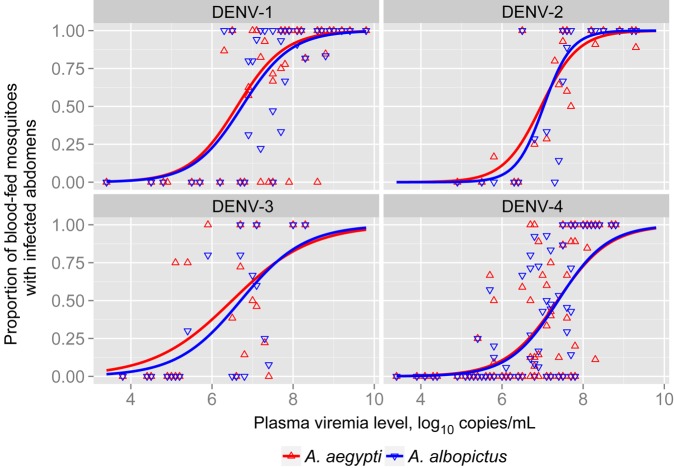
Dose-response scatterplot and curve (derived from logistic regression models) of plasma viremia versus the proportion of mosquitoes with dengue virus (DENV)–infected abdomens after feeding on 118 DENV-infected patients, showing curves for *Aedes aegypti* and *Aedes albopictus.* Each dot represents the proportion of mosquitoes that took a blood meal during an exposure event and had DENV-infected abdomens 14 days later and the corresponding plasma viremia level in the patient at the time of mosquito exposure, stratified by DENV serotype.

There was a dose-response relationship between plasma viremia levels at the time of mosquito feeding and the proportion of mosquitoes of either species with DENV-infected abdomens 14 days later (Figure 1). The overall DENV MID_50_ for *A. aegypti* was lower than that observed for *A. albopictus*, but this difference was not statistically significant (7.0 log_10_ copies/mL [95% CI, 6.77–7.23] vs 7.1 log_10_ copies/mL [95% CI, 6.9–7.3]; Table 2). Viral RNA concentrations of all 4 DENV serotypes were significantly higher in abdomen tissues from infected *A. albopictus* than in those from *A. aegypti* (Supplementary Table 1)*.* Given that *A. albopictus* accommodated higher DENV RNA concentrations once infected, we examined whether this could result in greater virus sequence diversity. As a test case, the consensus nucleotide sequence of the gene encoding DENV-1 envelope protein was determined directly by analysis of plasma specimens from 10 patients and abdominal tissues from 20 *A. aegypti* and 20 *A. albopictus* that had taken blood meals from these cases (ie, 2 *A. aegypti* and 2 *A. albopictus* per patient). The median number of nucleotide differences between the consensus sequences of the gene recovered from plasma specimens and the gene recovered from abdominal tissue was 9 (interquartile range [IQR], 4–15) for *A. aegypti* and 17 (IQR, 11–18) for *A. albopictus* (*P* = .02, by the Wilcoxon test [stratified by patient]; Supplementary Figure 2).

**Table 2.** 50% Mosquito Infectious Doses (MID_50_ Values) for *Aedes albopictus* and *Aedes aegypti* Abdomen Infection, by Dengue Virus (DENV) Serotype

Serotype	*A. aegypti*	*A. albopictus*	Absolute Difference	*P* Value
DENV-1	6.62 (6.10–7.13)	6.74 (6.27–7.21)	0.12 (−.11 to .35)	.29
DENV-2	6.96 (6.54–7.38)	7.03 (6.62–7.43)	0.07 (−.17 to .30)	.59
DENV-3	6.49 (5.69–7.30)	6.70 (5.86–7.56)	0.21 (−.39 to .82)	.49
DENV-4	7.37 (7.04–7.70)	7.38 (7.07–7.69)	0.01 (−.21 to .24)	.89
Overall	7.00 (6.77–7.23)	7.10 (6.90–7.30)	0.10 (−.04 to .24)	.15

MID_50_ values (defined as plasma viremia levels corresponding to a 50% probability of infection) were derived on the basis of marginal logistic regression coefficients for each mosquito type, and corresponding 95% confidence intervals were calculated using the delta rule.

### Acquisition of Infectiousness Among *A. aegypti* and *A. albopictus*

**Figure 2.**
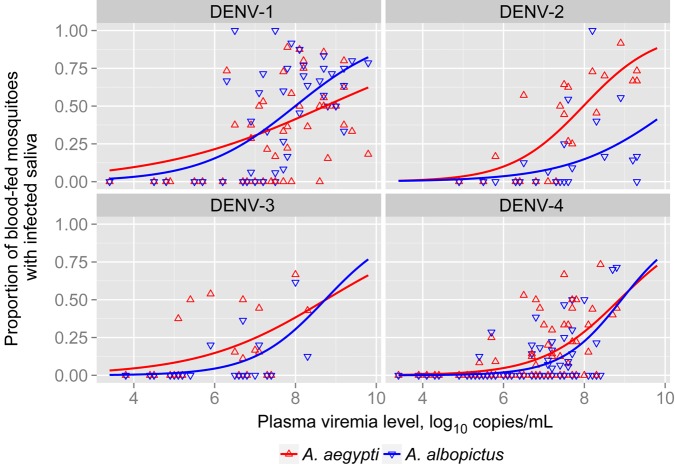
Dose response scatterplot and curve (derived from logistic regression models) of plasma viremia versus the proportion of mosquitoes with infectious saliva after feeding on 118 dengue virus (DENV)–infected patients, showing curves for *Aedes aegypti* and *Aedes albopictus.* Each dot point represents the proportion of mosquitoes that took a blood meal during an exposure event and had saliva containing infectious DENV 14 days later and the corresponding plasma viremia level in the patient at the time of mosquito exposure, stratified by DENV serotype.

As was observed in abdomen tissues, there was a dose-response relationship between the plasma viremia level and the proportion of mosquitoes with infectious saliva 14 days after blood feeding (Figure 2). However, the likelihood of detecting infectious saliva differed by mosquito species and DENV serotype. Table 3 summarizes the odds of abdomen and saliva infection and demonstrates that the detection of infectious saliva was less likely in blood-fed *A. albopictus*, compared with blood-fed *A. aegypti*, in both unadjusted analysis and analysis that adjusted for plasma viremia level (adjusted odds ratio [OR], 0.70; 95% CI, .52–.93). By serotype, the odds of *A. albopictus* having infectious saliva were significantly lower for blood meals involving uptake of DENV-2 and DENV-4, compared with those involving uptake of DENV-1 or DENV-3 (Table 3). These data identified the odds of *A. albopictus* becoming infectious as lower than the odds of *A. aegypti* becoming infectious after feeding on the blood of viremic patients. This analysis used all engorged mosquitoes as the denominator. Supplementary Table 2 shows the odds of having infectious saliva, using mosquitoes with DENV-positive abdomens as the denominator. This analysis confirms that the odds of *A. albopictus* becoming infectious were lower than those for *A. aegypti* after viremic blood feeding (OR, 0.69; 95% CI, .49–.96).

**Table 3.** Comparison of the Odds of Abdomen and Saliva Infection Among *Aedes albopictus* Versus the Odds Among *Aedes aegypti*, by Dengue Virus (DENV) Serotype

Specimen, Serotype	Patients, No.	*A. albopictus*	*A. aegypti*	Unadjusted OR (95% CI)	*P* Value	Adjusted OR (95% CI)^a^	*P* Value
		Infected/Tested, No. (%)	Infected/Tested, No. (%)				
Abdomen							
DENV-1	27	291/411 (70.8)	278/379 (73.4)	0.88 (.62–1.25)	.48	0.79 (.52–1.20)	.28
DENV-2	13	108/185 (58.4)	143/220 (65.0)	0.76 (.52–1.09)	.13	0.97 (.52–1.82)	.93
DENV-3	16	67/173 (38.7)	83/208 (39.9)	0.95 (.55–1.66)	.86	0.76 (.39–1.48)	.42
DENV-4	49	229/777 (29.5)	232/751 (30.9)	0.93 (.70–1.24)	.64	0.95 (.69–1.31)	.77
Overall	105	695/1546 (45.0)	736/1558 (47.2)	0.91 (.78–1.07)	.27	0.85 (.69–1.06)	.16
Saliva							
DENV-1	27	177/411 (43.1)	142/379 (37.5)	1.26 (.89–1.80)	.20	1.31 (.90–1.93)	.16
DENV-2	13	22/185 (11.9)	91/220 (41.4)	0.19 (.11–.33)	<.001	0.17 (.09–.31)	<.001
DENV-3	16	17/173 (9.8)	35/208 (16.8)	0.54 (.21–1.37)	.19	0.44 (.19–1.00)	.051
DENV-4	49	60/777 (7.7)	92/751 (12.3)	0.60 (.36–.99)	.044	0.60 (.37–.98)	.041
Overall	105	276/1546 (17.9)	360/1558 (23.1)	0.72 (.55–.95)	.022	0.70 (.52–.93)	.014

Abbreviations: CI, confidence interval; OR, odds ratio.

^a^ Marginal logistic regression models adjusted for plasma viremia.

### Covariates and Their Association With Successful Human-to-Mosquito Transmission

In multivariable regression analysis, a higher plasma viremia level at the time of exposure was independently associated with a greater likelihood of DENV transmission to abdominal tissue for both mosquito types. Each 1-log increase in the log_10_ plasma viremia level was associated with a 4.65-fold increase in the odds of abdominal tissue infection in *A. aegypti* (95% CI, 3.34–6.48) and a 5.3-fold increase in the odds of abdominal tissue infection in *A. albopictus* (95% CI, 3.80–7.43; Table 4). Each 1-log increase in the log_10_ plasma viremia level was associated with 2.16-fold increase in the odds of saliva infection in *A. aegypti* (95% CI, 1.68–2.78) and a 2.79-fold increase in the odds of saliva infection in *A. albopictus* (95% CI, 2.17–3.60; Table 4). These data highlight plasma viremia level as a risk factor for infectiousness among *A. aegypti* and *A. albopictus.* Supplementary Table 3 shows the association between these covariates and successful transmission, with mosquitoes with DENV-negative abdomens excluded from the model.

**Table 4.** Covariates and Their Association With Successful Dengue Virus (DENV) Transmission Among *Aedes albopictus* and *Aedes aegypti*

Variable	Abdomen				Saliva (Among All Mosquitoes )			
	*A. aegypti*		*A. albopictus*		*A. aegypti*		*A. albopictus*	
	Adjusted OR (95% CI)	*P* Value	Adjusted OR (95% CI)	*P* Value	Adjusted OR (95% CI)	*P* Value	Adjusted OR (95% CI)	*P* Value
Illness duration at enrollment (per 1-d increase)	0.58 (.36–.93)	.025	0.60 (.39–.92)	.018	0.87 (.61–1.24)	.45	0.91 (.62–1.34)	.63
Viremia level (per 1-log_10_ copies/mL increase)	4.65 (3.34–6.48)	<.001	5.32 (3.80–7.43)	<.001	2.16 (1.68–2.78)	<.001	2.79 (2.17–3.60)	<.001
DENV serotype								
DENV-1	1.00 (reference)		1.00 (reference)		1.00 (reference)		1.00 (reference)	
DENV-2	0.57 (.18–1.78)	.34	0.74 (.24–2.34)	.59	1.43 (.69–2.95)	.34	0.18 (.08–.39)	<.001
DENV-3	1.29 (.30–5.49)	.73	1.31 (.31–5.47)	.71	1.08 (.41–2.86)	.88	0.40 (.12–1.38)	.15
DENV-4	0.27 (.10–.71)	.008	0.36 (.15–.88)	.026	0.48 (.24–.99)	.046	0.23 (.12–.44)	<.001
Serological profile								
Primary	1.00 (reference)		1.00 (reference)		1.00 (reference)		1.00 (reference)	
Secondary	0.84 (.18–3.86)	.83	0.73 (.18–2.96)	.66	0.76 (.29–1.99)	.58	1.01 (.45.2.26)	.99
Indeterminate	0.27 (.06–1.16)	.08	0.51 (.11–2.43)	.40	0.37 (.13–1.09)	.07	0.31 (.09–1.02)	.054

Data are from marginal models.

Abbreviations: CI, confidence interval; OR, odds ratio.

## DISCUSSION

There is speculation on the arboviral disease risks posed by the invasion of *A. albopictus* into southern Europe and the United States [25]. Central to calibrating these risk assessments is an understanding of *A. albopictus* vector competence. There is conflicting literature on whether *A. albopictus* is less susceptible than *A. aegypti* to disseminated DENV infection [26–32]. Furthermore, all of the previous work in this area has used laboratory methods that do not mimic natural infection. Here, in experiments involving direct feeding on viremic patients with dengue, we provide evidence that *A. albopictus* had a susceptibility to acquiring DENV infection that was similar to that of *A. aegypti.* However, blood-fed *A. albopictus* was significantly less likely to become infectious with DENV-2 and DENV-4, suggesting an interspecies difference of epidemiological importance.

A first step in the establishment of DENV infection in mosquitoes is attachment of virus particles to luminal receptors on the midgut epithelium, followed by virus entry via endocytic pathways. There are few insights into the nature of these receptors for DENV, although it has been proposed that the R67 and R80 proteins may act as midgut receptors leading to subsequent systemic infection [33]. Our finding that the plasma viremia MID_50_ values for *A. aegypti* and *A. albopictus* were broadly similar suggests that DENV uses common mechanisms to attach and initially infect the midgut epithelium in these specimens. The MID_50_ values for DENV-1 and DENV-4 measured in this study were concordant with those observed previously, whereas the MID_50_ values differed for DENV-2 and DENV-3 [22]. We speculate that this reflects the smaller numbers of patients with DENV-2 and DENV-3 in this current study and hence decreased accuracy.

Our results identified that *A. albopictus* and *A. aegypti* were equally likely to develop infectious saliva containing DENV-1 and DENV-3. However, DENV-2 and DENV-4 were detected in saliva from *A. albopictus* less frequently than in saliva from *A. aegypti*. This might simply reflect intrinsic differences between the virus types in their virulence for *A. albopictus*, with DENV-1 and DENV-3 being fitter in this instance. However, it is interesting to note that, while DENV-1 was the dominant serotype in the Key West outbreak of dengue with *A. aegypti* as the vector, DENV-1 has not become further established in the southern United States, where *A. albopictus* is prevalent [34].

We explored the relationship between various covariates and the likelihood of successful human-to-mosquito transmission. We confirmed the importance of the plasma viremia level in successful human-mosquito transmission: each 1-log increase in plasma viremia level was associated with an approximately 5-fold increase in the odds of successful human-to-mosquito transmission for both *A. aegypti* and *A. albopictus* [22]. This finding again suggests that interventions that reduce DENV viremia during natural infection could have both an individual benefit and a public health benefit through reducing human infectiousness and thus reducing the risk of further transmission [19].

DENV RNA concentrations were significantly higher in abdominal tissues of *A. albopictus* than *A. aegypti* for all serotypes. When examined for DENV-1, this was also associated with higher levels of sequence drift away from the consensus sequence of the gene encoding envelope protein in the patient's plasma sample. Plausibly, by being more permissive to DENV replication in its abdominal tissues, *A. albopictus* might contribute to the genetic diversity that exists within DENV populations. Despite *A. albopictus* harboring higher viral burdens in abdominal tissues, significantly fewer *A. albopictus* had infectious saliva resulting from DENV-2 or DENV-4 infections. These data are broadly consistent with previous observations demonstrating that *A. albopictus* was more susceptible to fulminant DENV infection of the mosquito body but that viral dissemination to saliva was less than in *A. aegypti* [18]. That such nuanced interspecies differences can exist is perhaps not surprising, given the literature reporting variation in the susceptibility of *A. aegypti* populations to DENV infection, albeit under laboratory conditions [35–39]. Future studies to understand the basis for these phenotypes, particularly when using field-derived mosquitoes and direct feeding on human viremic hosts, will require large sample sizes to overcome the intrinsic variance in this system.

While our findings have identified interspecies differences in susceptibility to DENV infection, the absolute differences between the mosquito types are nonetheless small. This points to factors other than outright susceptibility to DENV infection as important reasons why *A. albopictus* is a marginal contributor to dengue transmission in urban and peri-urban settings in dengue-endemic countries. Behavioral attributes are also important for vector competence, and thus we speculate that the tendency of *A. aegypti* to live proximate to humans is the major reason why it is the primary DENV vector.

Our study has limitations [18]. First, for practical reasons we measured the phenotype of blood-fed, field-derived mosquitoes at 14 days after feeding; data acquisition at other time points might have led to additional insights. Second, the results were limited in scope to the virus serotypes and genotypes in circulation during the study period, and consequently we acquired sparse data on DENV-2 and DENV-3. Additionally, there may be specific interactions between DENV types and mosquito genotypes that influence infection outcome [40]. Studies in other settings should be encouraged to understand the generalizability of this work.

In summary, we are the first to demonstrate that *A. albopictus* are less likely than *A. aegypti* to develop an infectious phenotype 14 days after direct blood feeding on viremic patients with dengue due to DENV-2 or DENV-4 infection. These results will enable more-accurate parameterization of DENV transmission models in regions where dengue is endemic and those that are at risk for endemicity. In addition, we have confirmed the central importance of plasma viremia in determining the likelihood of mosquito tissue infection, suggesting that interventions that attenuate viremia will have both individual and community benefits.

## Supplementary Data

Supplementary materials are available at *The Journal of Infectious Diseases* online (http://jid.oxfordjournals.org). Supplementary materials consist of data provided by the author that are published to benefit the reader. The posted materials are not copyedited. The contents of all supplementary data are the sole responsibility of the authors. Questions or messages regarding errors should be addressed to the author.

Supplementary Data
