# Supplementary material for: Comparative Susceptibility of Aedes albopictus and Aedes aegypti to Dengue Virus Infection After Feeding on Blood of Viremic Humans: Implications for Public Health
Source: J Infect Dis. 2015 Mar 17;212(8):1182–90. doi: 10.1093/infdis/jiv173 (PMC4577038; doi:10.1093/infdis/jiv173)
Supplement: Supplementary Data [file supp_212_8_1182__index.html]

Comparative Susceptibility of Aedes albopictus and Aedes aegypti to Dengue Virus Infection After Feeding on Blood of Viremic Humans: Implications for Public Health — Comparative Susceptibility of Aedes albopictus and Aedes aegypti to Dengue Virus Infection After Feeding on Blood of Viremic Humans: Implications for Public Health — Supplementary Data 

# Comparative Susceptibility of *Aedes albopictus* and *Aedes aegypti* to Dengue Virus Infection After Feeding on Blood of Viremic Humans: Implications for Public Health

## Supplementary Data

Supplementary Data

**Files in this Data Supplement:**

- Supplementary Figure 2 - pdf file
- Supplementary Figure 1 - docx file
- Supplementary Table 1 - docx file
- Supplementary Table 2 - docx file
- Supplementary Table 3 - docx file
