# Supplementary material for: Comparative Susceptibility of Aedes albopictus and Aedes aegypti to Dengue Virus Infection After Feeding on Blood of Viremic Humans: Implications for Public Health
Source: J Infect Dis. 2015 Mar 17;212(8):1182–90. doi: 10.1093/infdis/jiv173 (PMC4577038; doi:10.1093/infdis/jiv173)
Supplement: Supplementary Data [file supp_jiv173_jiv173supp_fig2.pdf]

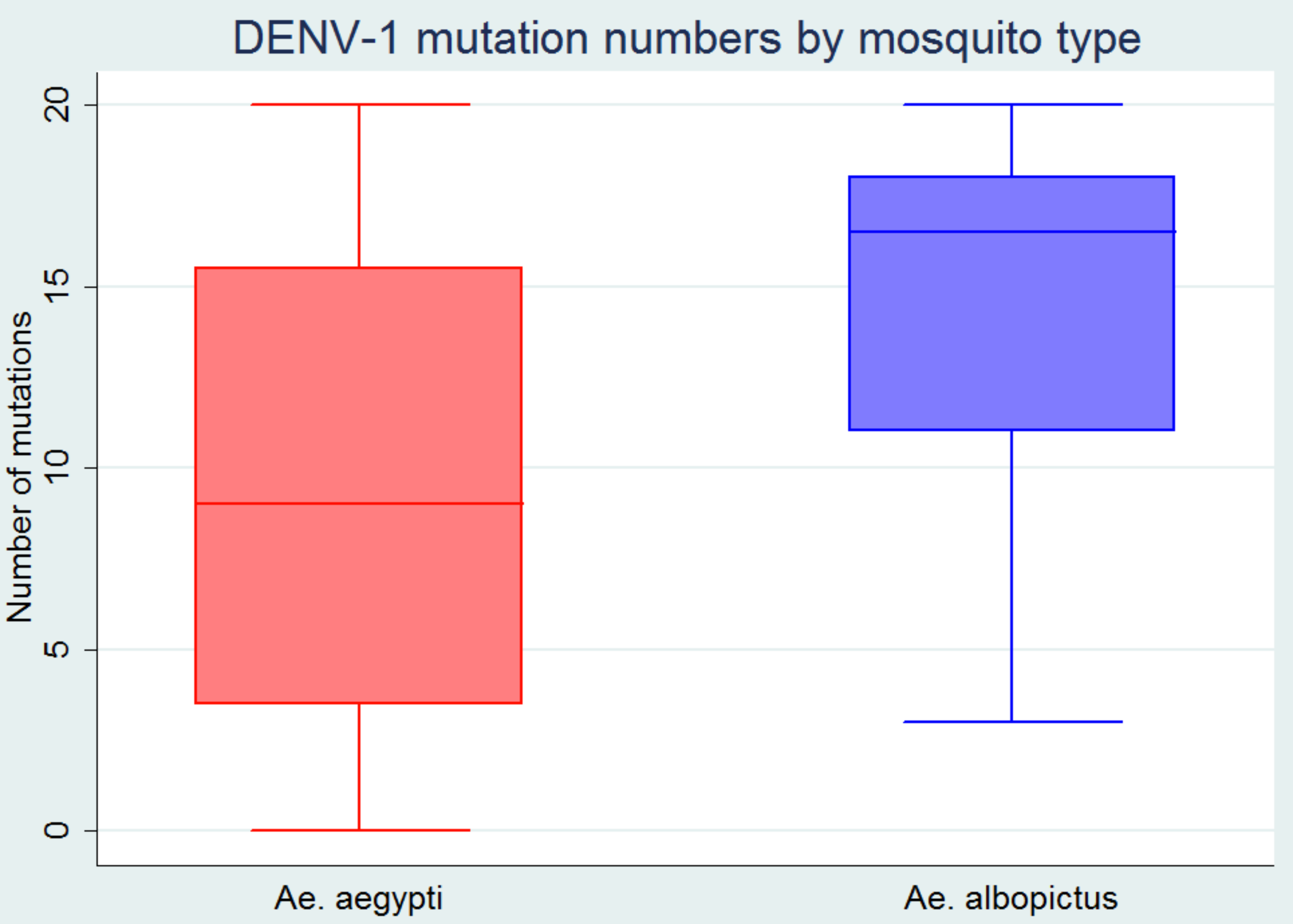
DENV-1 mutation numbers by mosquito type
Number of mutations
0
5
10
15
20
Ae. aegypti
Ae. albopictus

Figure S2: Box plot showing the number of mutations from baseline by mosquito type stratified by patient
