# Supplementary material for: Comparative Susceptibility of Aedes albopictus and Aedes aegypti to Dengue Virus Infection After Feeding on Blood of Viremic Humans: Implications for Public Health
Source: J Infect Dis. 2015 Mar 17;212(8):1182–90. doi: 10.1093/infdis/jiv173 (PMC4577038; doi:10.1093/infdis/jiv173)
Supplement: Supplementary Data [file supp_jiv173_jiv173supp_fig1.docx]

**Figure S1:** Flow chart of patient enrolment and exposure to mosquitoes. IQR, interquartile range

Enrolled: 120 (78%)

670 *Ae. aegypti* died

647 *Ae. albopictus* died

Final cohort for analysis: 118 patients with 232 exposure events for 1889 blood-fed *Ae. aegypti* and 1988 *Ae. albopictus*

Median number (IQR) engorged *Ae. aegypti* per event: 11 (8-13)

Median number engorged *Ae. albopictus* per event: 11 (8-14)

Total engorged *Ae. aegypti*: 2559

Total engorged *Ae. albopictus*: 2635

Withdrew: 6 (5%)

2 withdrew before exposure

118 patients exposed to a median number (IQR) of 15 (14-16) *Aedes aegypti* and 19 (17-20) *Aedes albopictus* on 232 occasions (114 exposed twice and 4 exposed once)

NS1 positive: 153 (50%)

Clinically suspected dengue cases: 306
