# Supplementary material for: Comparative Susceptibility of Aedes albopictus and Aedes aegypti to Dengue Virus Infection After Feeding on Blood of Viremic Humans: Implications for Public Health
Source: J Infect Dis. 2015 Mar 17;212(8):1182–90. doi: 10.1093/infdis/jiv173 (PMC4577038; doi:10.1093/infdis/jiv173)
Supplement: Supplementary Data [file supp_jiv173_jiv173supp_table1.docx]

|  | **Supplementary Table 1: Abdomen viral burden (log10 RNA copies per abdomen) according to mosquito type *** | | | | | | |
| --- | --- | --- | --- | --- | --- | --- | --- |
|  | | **Serotype** | **Number of Ae. aegypti** | **Ae. aegypti**  **(median, IQR)** | **Number of Ae. albopictus** | **Ae. albopictus**  **(median, IQR)** | **p value** |
|  | | All | 1472 | 7.3(6.9-7.6) | 1390 | 7.7(7.4-8.0) | <0.0001 |
|  |  | DENV 1 | 556 | 7.0(6.7-7.3) | 582 | 7.6(7.4-7.9) | <0.0001 |
|  |  | DENV 2 | 286 | 7.5(7.2-7.8) | 216 | 7.8(7.4-7.9) | <0.0001 |
|  |  | DENV 3 | 166 | 7.1(6.6-7.5) | 134 | 7.4(7.1-7.7) | <0.0001 |
|  |  | DENV 4 | 464 | 7.4(7.2-7.7) | 458 | 7.8(7.6-8.1) | <0.0001 |
|  | *Among infected abdomen tissues only. | | | | | | |
|  | IQR: Interquartile range; p value from Wilcoxon rank-sum tests | | | | | | |
