# Supplementary material for: Comparative Susceptibility of Aedes albopictus and Aedes aegypti to Dengue Virus Infection After Feeding on Blood of Viremic Humans: Implications for Public Health
Source: J Infect Dis. 2015 Mar 17;212(8):1182–90. doi: 10.1093/infdis/jiv173 (PMC4577038; doi:10.1093/infdis/jiv173)
Supplement: Supplementary Data [file supp_jiv173_jiv173supp_table2.docx]

**Supplementary Table 2. Saliva infection among positive abdomen**

|  | **No of patients** | **Ae. albopictus** | | **Ae. aegypti** | | **Unadjusted OR (95CIs)** | **p-values** | **Adjusted OR (95CIs)*** | **p-values** |
| --- | --- | --- | --- | --- | --- | --- | --- | --- | --- |
|  |  | **# infected/ N abdomen positive** | **%** | **# infected/ N abdomen positive** | **%** |  |  |  |  |
| DENV1 | 27 | 177/291 | 60.8 | 142/278 | 51.1 | **1.49(1.01-2.19)** | **0.045** | **1.49(1.01-2.19)** | **0.046** |
| DENV2 | 13 | 22/108 | 20.4 | 91/143 | 63.6 | **0.15(0.08-0.27)** | **<0.001** | **0.15(0.08-0.28)** | **<0.001** |
| DENV3 | 16 | 17/67 | 25.4 | 35/83 | 42.2 | 0.49(0.23-1.06) | 0.07 | **0.45(0.24-0.85)** | **0.014** |
| DENV4 | 49 | 60/229 | 26.2 | 92/232 | 39.7 | **0.54(0.30-0.96)** | **0.037** | **0.55(0.31-0.97)** | **0.040** |
| All | 105 | 276/695 | 39.7 | 360/736 | 48.8 | **0.69(0.49-0.98)** | **0.035** | **0.69(0.49-0.96)** | **0.030** |
| *Marginal logistic regression models for adjusted for plasma viremia | | | | | | | | | |
