# Supplementary material for: Comparative Susceptibility of Aedes albopictus and Aedes aegypti to Dengue Virus Infection After Feeding on Blood of Viremic Humans: Implications for Public Health
Source: J Infect Dis. 2015 Mar 17;212(8):1182–90. doi: 10.1093/infdis/jiv173 (PMC4577038; doi:10.1093/infdis/jiv173)
Supplement: Supplementary Data [file supp_jiv173_jiv173supp_table3.docx]

|  | **Saliva (among positive abdomen)** | | | |
| --- | --- | --- | --- | --- |
| **Variable** | **Aegypti** | | **Albopictus** | |
|  | **Adjusted OR(95%CI)** | **p value** | **Adjusted OR(95%CI)** | **p value** |
| Day of illness at enrolment (+1 day) | 0.99(0.73-1.34) | 0.95 | 1.07(0.78-1.46) | 0.70 |
| Viremia (+1 log -copies/ml) | 1.01(0.78-1.31) | 0.95 | **1.57(1.20-2.05)** | **<0.001** |
| Serotype |  |  |  |  |
| DENV1 | 1.00(reference) |  | 1.00(reference) |  |
| DENV2 | 1.68(0.93-3.01) | 0.08 | **0.17(0.08-0.35)** | **<0.001** |
| DENV3 | 0.65(0.31-1.36) | 0.26 | **0.31(0.11-0.91)** | **0.032** |
| DENV4 | 0.62(0.34-1.11) | 0.10 | **0.29(0.16-0.51)** | **<0.001** |
| Serology |  |  |  |  |
| Primary | 1.00(reference) |  | 1.00(reference) |  |
| Secondary | 0.91(0.49-1.71) | 0.77 | 1.11(0.63-1.95) | 0.72 |
| Indeterminate | 0.74(0.32-1.73) | 0.49 | **0.42(0.17-0.99)** | **0.049** |

**Supplementary Table 3. Covariates and their association with successful DENV transmission among positive abdomen**
